# Feedback on end-of-life care in dementia: the study protocol of the FOLlow-up project

**DOI:** 10.1186/1472-684X-12-29

**Published:** 2013-08-07

**Authors:** Jannie A Boogaard, Mirjam C van Soest-Poortvliet, Johannes R Anema, Wilco P Achterberg, Cees M P M Hertogh, Henrica C W de Vet, Jenny T van der Steen

**Affiliations:** 1 Department of General Practice & Elderly Care Medicine, EMGO Institute for Health and Care Research, VU University Medical Center, Van der Boechorststraat 7, 1081BT, Amsterdam, The Netherlands; 2 Gerion, VU University Medical Center, Amsterdam, The Netherlands; 3 Department of Public and Occupational Health, EMGO Institute for Health and Care Research, VU University Medical Center, Amsterdam, The Netherlands; 4 Department of Public Health and Primary Care, Leiden University Medical Center, Leiden, The Netherlands; 5 Department of Epidemiology and Biostatistics, EMGO Institute for Health and Care Research, VU University Medical Center, Amsterdam, The Netherlands

**Keywords:** End-of-life care, Satisfaction with care, Quality of care, Dementia, Nursing home, Quality indicators

## Abstract

**Background:**

End-of-life care in dementia in nursing homes is often found to be suboptimal. The Feedback on End-of-Life care in dementia (FOLlow-up) project tests the effectiveness of audit- and feedback to improve the quality of end-of-life care in dementia.

**Methods/Design:**

Nursing homes systematically invite the family after death of a resident with dementia to provide feedback using the End-of-Life in Dementia (EOLD) – instruments. Two audit- and feedback strategies are designed and tested in a three-armed Randomized Controlled Trial (RCT): a generic feedback strategy using cumulative EOLD-scores of a group of patients and a patient specific feedback strategy using EOLD-scores on a patient level. A total of 18 nursing homes, three groups of six homes matched on size, geographic location, religious affiliation and availability of a palliative care unit were randomly assigned to an intervention group or the control group. The effect on quality of care and quality of dying and the barriers and facilitators of audit- and feedback in the nursing home setting are evaluated using mixed-method analyses.

**Discussion:**

The FOLlow-up project is the first study to assess and compare the effect of two audit- and feedback strategies to improve quality of care and quality of dying in dementia. The results contribute to the development of practice guidelines for nursing homes to monitor and improve care outcomes in the realm of end-of-life care in dementia.

**Trial registration:**

The Netherlands National Trial Register (NTR). Trial number: NTR3942

## Background

The prevalence of dementia worldwide is significantly growing, with the majority of the persons with dementia dying in nursing homes [1,2]. Therefore, the provision of high-quality end-of-life care for nursing home residents with dementia is essential [3-6]. However, the literature reports numerous shortcomings in the end-of-life care for dementia, suffering of residents and unfulfilled needs of families [6]. For example, an Italian study reported high levels of pressure ulcers, burdensome interventions such as tube- and PEG-feeding, psychotropic drugs and poor decision-making in the last month of life of nursing home residents with dementia [7]. Despite some encouraging trends from The Netherlands and the U.S. regarding improved symptom management in dementia [8-10], improvement of end-of-life care for dementia remains a research priority [11].

Systematic assessments of care performance that are compared to professional targets or standards (hereafter referred to as audit and feedback) is widely used as a strategy to improve professional care practice [12]. In the nursing home setting, there are indications that audit and feedback using cumulative quality of care scores based on a group of patients may improve nursing home care in general [13-15], including nursing home care for residents with dementia [16]. In the US, audit and feedback is already structurally applied to improve hospice and palliative care services (Family Evaluation of Hospice Care Survey of the National Hospice and Palliative Care Organization, [17]).

The literature suggests that audit and feedback is more effective when accompanied by either active interventions (such as educational outreach, integration within an overall quality improvement framework), or passive interventions (such as publication of performance), with active interventions generally being more successful then passive interventions [15,18-20]. So far, only audit- and feedback strategies using cumulative scores relating to care performances of care teams have been reported previously in the literature (e.g., Zuidgeest et al. [21]). However, this audit- and feedback strategy is time consuming due to the administrative tasks involved, which potentially creates barriers for the nursing homes to use audit- and feedback for care quality improvement. Therefore, a feedback strategy based on discussing evaluations on a patient level, is an appealing, and possibly less time consuming, alternative design. Such patient specific audit- and feedback also allows for individual care workers to relate more directly the feedback to their own care performance.

Due to a lack of studies that directly compare different strategies of audit and feedback, evidence for the effectiveness of different audit and feedback strategies is limited [15,19], and this includes the nursing home setting. Moreover, the influence of the organizational context on audit- and feedback and its implementation has not been addressed. More generally, earlier work in the area of evidence-based clinical practices in health care organizations found three organizational elements to influence implementation processes of evidence-based clinical practices: active leadership, process adaptation and involvement of management structures and processes [22]. Implementation of guidelines is affected by the specific characteristics of the guidelines, the target group and of the social or environmental context [23].

The aim of the Feedback on End-of-Life care in dementia (FOLlow-up) project is to assess the effect of the implementation of two audit- and feedback strategies on the quality of care and quality of dying of nursing home residents with dementia: a generic feedback strategy using cumulative care performance scores generated by a feedback program, and a patient specific strategy. Effects of implementation are assessed with the End-of-Life in Dementia – Satisfaction With Care (EOLD-SWC) scale and the End-of-Life in Dementia – Comfort Assessment in Dying (EOLD-CAD) scale [24]. Families evaluate and provide feedback on the quality of end-of-life care and the quality of dying of nursing home residents with dementia, as families’ perceptions are intrinsically valuable in palliative care [25]. These instruments had the best psychometric properties and feasibility for use among bereaved family members [26-28]. Further, this study improves our understanding of facilitators and barriers of implementation, and of effectiveness to improve care of audit and feedback in the nursing home setting using a mixed-method process evaluation.

## Methods

### Study design

The effects of active implementation of the EOLD-instruments is tested using a Randomized Controlled Trial (RCT) design. Nursing homes are randomized into three groups. Two intervention groups implement the EOLD-instruments according to the generic or the patient-specific feedback strategy, and a control group is created to control for changes that occur over time in the nursing home setting (2005–2010) independent from feedback on quality of care [9].

### Setting and study population

Participating nursing homes implement the EOLD-SWC and EOLD-CAD instruments on psychogeriatric wards (almost all dementia, and patients generally stay there until death). A specially trained elderly care physician employed by the nursing home is responsible for the care, including the residents’ last stage of life [29-31].

The study population comprises family caregivers (i.e., the main contact person) of nursing home residents with dementia who died on a psychogeriatric ward. Families of residents who stayed at least 16 days of the last month of their life in the nursing home are eligible to provide written feedback. Further, potential respondents need to be able to read Dutch. The nursing home invites the family member most involved in care during the last month (usually the same person throughout admission) to provide feedback.

### Power analyses and recruitment of nursing homes

The power analyses were based on a minimum number of family assessments to generate feedback; from there, we calculated the number of facilities in each group, from which followed a minimum and average number of beds per facility. For the cumulative feedback strategy, a minimum of 10 to 15 feedback reports is required to generate reliable total EOLD-SWC and EOLD-CAD scores and compare with national means, and we departed from an average total of 30 feedback reports for the complete data collection period. Further, the minimum relevant difference to be detected on the EOLD instruments before and after implementation of the feedback was 3 points. Based on three previous Dutch studies using the EOLD-SWC and EOLD-CAD instruments, we assumed an Intra Class Correlation Coefficient of 0.07 for the EOLD-CAD and 0.01 for the EOLD-SWC [9]. Additionally, when taking into account a significance level (alpha) of 0.05 and a power (beta) of 0.80, a minimum of five nursing homes per intervention group is needed. Based on a rate of 55% for eligibility and response, each participating nursing home needs to have a minimum of 22 decedents with dementia per year, and the average across facilities should amount to 33. Assuming a quarter of the nursing home residents die each year [32], the minimum number of beds of the psychogeriatric wards of participating nursing homes is 88, and the average over all facilities should amount to 132.

Nursing homes meeting the criterion of the availability of a minimum of around 88 psychogeriatric care beds have been recruited from all over the country. Nursing homes that were planning an organizational change that might affect the study’s outcomes were excluded from participation. Fifty-six nursing homes with the required number of psychogeriatric beds located throughout the country have been approached to be involved in the study. From the approached nursing homes, two nursing homes could not participate due to the exclusion criteria. A total of 18 nursing homes agreed to participate in the study (recruitment rate: 32%). The most common reasons not to participate were lack of time, organizational changes or staff shortage, and nursing homes not having end-of-life care quality improvement as their current priority.

### Randomisation

Based on the variability in factors potentially affecting resident outcome and family satisfaction with care as reported in the literature (reviewed by Van der Steen, 2013 [32]), three groups were matched to ensure similar distributions with regard to the following characteristics: size, geographic location, religious affiliation and the availability of a palliative care unit, since a spill-over effect of hospice services on residents who were not on hospice has been noted. Subsequently, the three groups were randomly assigned to one of the two intervention groups or the control group.

### The intervention

#### Theoretical framework and hypotheses

**Figure 1:**
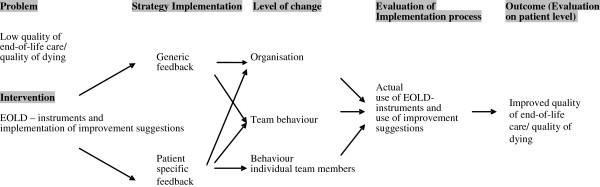
Conceptual model for effectiveness of two feedback strategies.

The FOLlow-up project aims at changing the behavior of professional caregivers on different levels in the nursing home due to the implementation of the EOLD-instruments in the nursing home practice (Figure 1). We hypothesize that informing nursing homes on their cumulative EOLD-scores using the generic feedback strategy linked to identified care deficits will motivate nursing homes to improve both as an organization and as a care team. Similarly, we assume that patient specific feedback may, in addition to changes in care performance on an organizational level and team level, result in behavioral changes of an individual professional caregiver. For example, if a physician received feedback from a family that the explanation of medication issues was unclear, he may improve the informing about medication to family members. Further, discussing of this in the care team possibly has a spin-off to practice of colleagues, which may result in standard offering of an information leaflet on selected medication.

### The EOLD- instruments

Earlier research reviewed eleven measurement instruments developed to assess the quality of end-of-life care and quality of dying of nursing home residents with or without dementia. The End-of-Life in Dementia-Satisfaction With Care scale (EOLD-SWC) and the End-of-Life in Dementia-Comfort Assessment in Dying scale (EOLD-CAD) were identified as the most appropriate instruments with regard to, for example, validity, reliability, and ease of use, to assess quality of end-of-life care and quality of dying in dementia, respectively [26-28].

The EOLD–SWC is a 10-item scale that was developed for after-death assessment of satisfaction with care by family members of residents with dementia. Examples of items are ‘I felt fully involved in all decision making’ or ‘The health care team was sensitive to my needs and feelings.’ For both scales, higher scores reflect higher levels of comfort and higher levels of satisfaction respectively. The EOLD–CAD is a 14-item scale developed to assess the condition of the care recipient during the dying process. The scale comprises the subscales physical distress, dying symptoms, emotional symptoms, and well-being [24].

### Data collection and procedures

We also ask families to provide socio-demographic characteristics of both the respondent (age, gender, marital status, relationship to the nursing home resident) and of the decedent (age, gender, marital status and date of death).

The participating nursing homes send the questionnaire with the EOLD-instruments to the family caregiver of a nursing home resident who died with dementia. During 20 months the deaths on the nursing homes’ psychogeriatric wards are recorded. Six to eight weeks after the death of their loved one, the nursing home sends the questionnaire to the family caregivers. Along with the questionnaire, the family caregivers receive a letter that explains the involvement of the nursing home in the FOLlow-up project, and the returning of a completed questionnaire is considered as informed consent to participate. Further, the exact dates on which the questionnaires were sent out and received back, as well as the number of residents with dementia who died and whose family caregiver could not provide written feedback, and the reasons for ineligibility are registered. It is up to the nursing home to decide which staff member is most eligible to be responsible for the registration and sending of the questionnaires, but usually these tasks are performed by a member of the nursing homes’ administrative support team.

### Strategies for implementation

The two strategies to implement the EOLD-instruments 1) the generic feedback strategy and 2) the patient specific feedback strategy both link to specific suggestions on how to improve care. The improvement suggestions were developed based on the latest national and international literature and care guidelines in the field of end-of-life- and palliative care, and when available, specific to dementia [6,18,33-37]. They also included practical suggestions to inspire improvements even in the absence of evidence. Subsequently, the improvement suggestions were reviewed by professionals in the field on their practical applicability to improve care quality. Table 1 provides an example of an item of the EOLD-SWC scale with the related suggestion for care improvement.

**Table 1:** Example of an EOLD- SWC item with improvement suggestions

**End-of-life in dementia – satisfaction with care (EOLD-SWC) scale, item 7**	
**I feel that my relative/care recipient got all necessary nursing assistance**	
**Improvement suggestions (version 2.0):**	**Involved disciplines:**
a) Make clear to the family caregivers what the possible options are for nursing assistance for their relative with dementia. To support information provision, a booklet with information regarding nursing assistance in the last stage of life of residents with dementia may be handed out.	Physicians and nurses/nurse aides
b) In communication with family caregivers, you may wish to be realistic about the prognosis of their relative with dementia. Provide contact details of the staff members with whom family caregivers may talk with regard to the prognosis of their relative and its risks.¹	Physicians and nurses/nurse aides
c) Evaluate frequently (at least once in six months) in multi-disciplinary team meetings whether all possible nursing assistance is provided to the residents with dementia.	Physicians and nurses/nurse aides

¹To feel home. [Guidance for family caregivers and nursing home staff to work together to provided care with dignity], 2009.

The generic feedback strategy links cumulative EOLD-scores to specific targets to improve care quality. For this, a user-friendly import program has been developed for nursing homes to enter their EOLD item scores and generate total EOLD scores after the scores of at least ten residents are entered. The total EOLD-scores are compared with a norm based on mean EOLD item- and total scores collected nation wide in nursing homes using family caregivers’ evaluations of quality of care and quality of dying. The scores that are significantly higher or lower than the national mean item- and total scores are signaled. The program links to improvement suggestions tailored to the specific areas where the nursing home scored significantly lower, to trigger actions for care quality improvements.

In the patient specific strategy, individual patient EOLD-item scores are discussed in multi-disciplinary team meetings. To support the team discussions, the nursing homes using the patient-specific strategy will receive a printed version of all the improvement suggestions. The nursing homes of the intervention groups report the improvement actions initiated after receiving feedback to improve care quality.

### Evaluation of the FOLlow-up project

The effect of active implementation of the EOLD-instruments on quality of care is tested with a quantitative effect evaluation. Further, to assess the impact of the implementation of the instruments in the nursing homes, a process evaluation is performed. The development of the instrument for evaluation is informed by pilot work, exploring receptiveness of nursing homes to employ the EOLD-instruments. A pilot survey study among 40 Dutch nursing homes assessed their willingness to use these instruments in their daily psycho-geriatric practice as well as barriers and facilitators for effective use of the EOLD-instruments for care quality improvement. From the surveyed nursing homes, 63% would be willing to use the instruments. Their main motivation was the wish to understand the quality of care they provided and the possibility to improve this. The barriers named by the nursing homes were the expected additional workload and time investment. Involvement of the nursing home staff, varying from the nursing homes’ management to the care staff, as well as grassroot support from the field and incorporation in the care quality framework were named as important facilitators for effectiveness of the instruments for quality improvement. From this pilot we learned that some support and guidance may be needed for successful implementation. Therefore, we aim at testing effects of an intervention that is sustainable with limited external support.

### Effect evaluation

Starting the first of May 2012, the nursing homes of all three groups administer the EOLD-instruments for the complete period of data collection. After 10 months, the nursing homes of the two intervention groups actively deploy the feedback with the help of the improvement suggestions for care quality improvement according to the audit and feedback strategy they were randomly assigned to. The nursing homes report to the research team the improvement actions that were initiated following the feedback. After having received the feedback, the nursing homes of the two intervention groups continue to administer the EOLD-instruments for another 10 months, along with using the improvement suggestions. The nursing homes of the control group will administer the EOLD-instruments during the full data collection period of 20 months while providing their usual care. After the data collection, those nursing homes will receive their EOLD-scores along with suggestions to improve care quality as well as support to implement improvements actions among the care teams similar to the nursing homes participating in the intervention groups.

The participating nursing homes are responsible for the collection of data, with limited support from the research team. The support of the research team comprises instruction meetings with nursing home staff involved in the data collection, written instruction material and regular contact by telephone or email.

### Statistical analysis

To compare the data longitudinally within and between the intervention groups and control groups, the participating nursing homes hand over the questionnaires they receive back from the families to the research team to enter the data in SPSS. Subsequently, we explore any changes over time in all three research groups, and in the intervention groups over the two periods of data collection separately. In all research groups, the EOLD-scores collected during the pre- and post-test data collection are compared per home and over all homes with paired tests. If needed due to changes over time related to, for example, a general trend or an increased focus on end-of-life care related to study participation, time dependent analyses are performed to control for the changes over time.

### Process evaluation

All participating nursing homes regardless their research group are invited for a mixed-method process evaluation after 10 months and after 20 months from the start of the data collection. The Linnan and Steckler [38] framework is used to guide the process evaluation. To assess the dose delivered and dose received, a written survey is administered to collect information regarding the number of surveys that were sent out, and the number of surveys that the nursing home received back. Further, nursing home representatives are asked to estimate additional time- and financial investments. Second, to assess the fidelity (i.e., whether the intervention was implemented as intended) and the facilitators and barriers of audit- and feedback in the nursing homes using the EOLD-instruments, a qualitative interview with the nursing home staff involved in data collection (such as the elderly care physician, management, administrative support) is performed. These interviews are transcribed verbatim, coded by more than one member of the research team, and themes defined. The information collected in the interviews is compared with the nursing homes’ own registrations of the dose delivered and dose received and logs of their time- and material investments.

Last, the reports of the nursing homes with respect to the improvement actions initiated will be analyzed. Each reported improvement action will be categorized in whether it aims a behavioral change of an individual professional caregiver, of a team of professional caregivers or a change on the organizational level (Figure 1).

#### Ethical considerations

The study protocol was approved by the Medical Ethics Committee of the VU University Medical Center. The research group receives coded family evaluations from the participating nursing homes with the key to remain in the nursing home.

## Discussion

The FOLlow-up project is, to the best of our knowledge, the first study to implement and compare audit-and feedback strategies in the nursing home setting specifically to improve end-of-life care in dementia. We assume the implementation of audit- and feedback in the nursing home to be a complex process involving multiple processes of care in the nursing home (e.g., care quality coordination, administrative support, management structures and multi-disciplinary care giving). The assessment of the effects of audit- and feedback on care quality using a RCT combined with the evaluation of organizational and social elements possibly influencing audit and feedback will contribute to its theoretical understanding and practical lessons for future implementation in nursing homes. Further, our study will advance our understanding of how to monitor care outcomes in the realm of end-of-life care in dementia. Indeed, the EOLD-instruments showed a positive trend in EOLD- scores over time [9] and differences in EOLD-scores were found between countries [39,40]. Our data will increase the understanding of the differences between nursing homes in quality of care and quality of dying using EOLD-scores, as well as the possibilities of the nursing home care staff to influence them. This knowledge may provide an evidence base for the development of quality indicators needed to systematically improve end-of-life care in dementia. Nevertheless, the design of the study involved a few important choices with regard to the development of the audit- and feedback strategies, the research setting and the data collection.

First, regarding the design of the audit- and feedback strategies, to develop a care standard needed for the feedback program used in the generic feedback strategy, a norm was created based on data collected in previous Dutch research [9]. In the data, mean satisfaction with care (EOLD-SWC) scores did not significantly vary across different geographic areas, although slightly lower mean scores for quality of dying (EOLD-CAD) were found for densely versus less densely populated areas. Nevertheless, we wish to employ a single national standard of quality, and single mean score for both instruments for any region in the Netherlands was the norm. Because of trends in time, it is important to continue monitoring EOLD scores, and we may consider an update of the norm using the pre-test data collected in FOLlow up, if we find important differences from the existing norm.

Second, we have no data on which to base an estimate of the response rate with patient specific feedback strategy. Based on previous Dutch research using coded data without names [9,27,32,41], we assumed in our power calculation, a response rate of 55-60% and a few cases being ineligible. In the FOLlow-up project, the participating nursing homes are fully responsible for the data collection process. Nursing homes directly communicate with the family caregivers to obtain their care evaluations. This avoids asking family caregivers’ consent to participate prior to sending the EOLD-instruments and they may feel that their privacy is better guaranteed, compared to data collection that involves contact with the University. We expect that because of this protocol for data collection, family caregivers will be more forthcoming with their feedback compared to our previous research, potentially increasing the response rate. However, in the patient-specific feedback strategy, family caregivers will be explicitly asked permission allowing their feedback to be discussed non-anonymously in a multidisciplinary team meeting. This may lead to a different response rate between the two audit- and feedback strategies due to family caregivers being more hesitant to participate in the patient-specific strategy compared to the generic strategy.

Third, with respect to choice of research setting, previous research performed on psychogeriatric units (for dementia) of residential care homes found the level of comfort assessed with the CAD-EOLD to be lower than in nursing homes [9]. Therefore, implementation of the EOLD-instruments in residential care homes potentially involves more significant care improvements, if potential barriers such as less physician oversight or leadership can be addressed. Nevertheless, due to the small size of psychogeriatric units in residential care homes in the Netherlands (typically around 20 beds) compared to nursing homes and the absence of an in-home elderly care physician, we test the effectiveness of the EOLD-instruments only in nursing homes.

Despite the benefits of giving nursing homes responsibility for the data collection, it may also negatively influence the outcomes of the project. The research team cannot fully control the protocols and implementation of the audit- and feedback strategies as intended. However, only by giving nursing homes responsibility over the data collection is it possible to evaluate the practical implications and effect of audit- and feedback on quality of care and quality of dying in dementia. Further, the nursing homes receive limited, but continuous support during the data collection by the researchers. When audit- and feedback is proven effective in improving the quality of care in dementia, our findings may be implemented on a larger scale, along with specific recommendations for effective implementation of audit- and feedback in nursing homes.

## Competing interests

The authors declare that they have no competing interests.

## Authors’ contributions

All authors have made substantial contributions to conception and design of the study. JAB, MvS-P, HCWdV and JTvdS have drafted the manuscript. All authors have revised it critically for important intellectual content and have given final approval of the version to be published.

## Pre-publication history

The pre-publication history for this paper can be accessed here:

http://www.biomedcentral.com/1472-684X/12/29/prepub

## Previous publications

Abstract submitted to the annual congress of Alzheimer Europe in October 2012. The abstract is available on the internet site of Alzheimer Europe.
